# Pleural effusion from intrathoracic migration of a ventriculo-peritoneal shunt catheter: pediatric case report and review of the literature

**DOI:** 10.1186/s13052-018-0480-2

**Published:** 2018-03-27

**Authors:** Federica Porcaro, Emidio Procaccini, Maria Giovanna Paglietti, Alessandra Schiavino, Francesca Petreschi, Renato Cutrera

**Affiliations:** 1 Academic Department of Pediatrics, Bambino Gesù Children’s Hospital, IRCCS, Rome, Italy; 2 Department of Neuroscience, Bambino Gesù Children’s Hospital, IRCCS, Rome, Italy

**Keywords:** Hydrocephalus, Ventriculoperitoneal shunt, Pleural effusion, Children

## Abstract

**Background:**

Pleural effusion is a rare complication of ventriculo-peritoneal (VP) cerebrospinal fluid (CSF) shunting and its diagnosis is difficult in patients with neurological and consciousness impairment.

**Case report:**

Herein we report the case of a child affected by Pfeiffer syndrome and hydrocephalus, shunted at the age of 3 months, who developed acute respiratory failure due to a right-sided pleural effusion 2 years later. Plain chest radiographs and computed tomography (CT) showed the intrathoracic migration of the right VP shunt abdominal tip. Beta-2 transferrin, a marker for CSF, was found in the pleural fluid and the hypothesis of a CSF hydrothorax was confirmed. Effusion was treated with a thoracentesis. Seven days after, the right VP shunt was revised; a ventriculo-atrial (VA) shunt was also placed on the left side to serve as the main CSF shunt and to prevent the recurrence of hydrothorax. We review the pediatric cases of CSF hydrothorax reported in the literature and discuss the mechanisms underlying this complication together with the possible treatments.

**Conclusion:**

Pleural effusion due to VP shunt insertion is a rare and potentially life-threatening condition that should be suspected in any patient with a VP shunt and respiratory failure. Signs of hydrothorax may moreover represent the only clinical evidence of a shunt-related complication in case of neurologically severely compromised patients in which neurologic examination cannot help to make a diagnosis.

**Electronic supplementary material:**

The online version of this article (10.1186/s13052-018-0480-2) contains supplementary material, which is available to authorized users.

## Background

Cerebrospinal fluid (CSF) shunting is still to be considered one of the main treatment of hydrocephalus, especially for infants affected by communicant hydrocephalus. Mechanical failures and infections are the most common shunt-related complications [1]. Thoracic complications of ventriculo-peritoneal (VP) shunts have rarely been reported and include pleural effusion, bronchial perforation, pneumothorax and pneumonia [2]. They may occur at any period of time after surgery. Three possible mechanisms have been postulated: intrathoracic trauma during shunt placement, dislocation of the peritoneal catheter into the chest and fluid shift from the peritoneal cavity to the pleural space [3].

CSF hydrothorax with shunt displacement can occur through congenital hiatuses or other small congenital defects in the diaphragm; erosion and perforation of the diaphragm due to inflammatory processes can also allow CSF to pass into the pleural cavity [1].

We report the case of a child affected by Pfeiffer syndrome with a shunted hydrocephalus who developed a right sided pleural effusion due to intrathoracic migration of the abdominal tip of the VP shunt. A review of pediatric cases of CSF hydrothorax in VP shunted patients is also provided.

## Case presentation

**Fig. 1:**
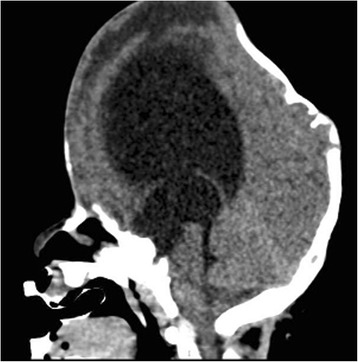
Sagittal MR image shows marked ventricular dilatation in our patient affected by Pfeiffer syndrome

A 2-year-old Caucasian female patient affected by Pfeiffer syndrome (de novo heterozygous mutations in FGFR2) and cloverleaf skull developed hydrocephalus (Fig. 1) and underwent a CSF shunting procedure (right VP shunt with a proximal programmable valve) at the age of 3 months. G-tube for dysphagia and tracheostomy for upper airway obstruction due to midface hypoplasia without other airway abnormalities were also needed. At the age of 9 months a fronto-orbital advancement with temporo-parietal osteotomies and cranial remodeling was carried out together with a permanent tarsorrhaphy.

She was admitted to our Unit for irritability, tachypnoea needing mechanical ventilation and increased baseline oxygen requirement of about 4 l per minute (LPM). She was afebrile and cardiovascular examination was unremarkable. Normal lung sounds were detected on thorax auscultation and good ventilation was noted bilaterally; no dullness to percussion was reported. Her abdomen was soft, non-distended and non-tender. Her neurological state, characterized by marked neurocognitive impairment, didn’t change and the permanent tarsorrhaphy made eyes’ position and pupillary reflexes’ evaluation impossible. The remainder of physical examination was unremarkable.

At the time of admission, results of venous blood gas and routine blood tests, including kidney and liver function, were normal. Although thorax auscultation was negative, described symptoms led us to request chest X-rays showing a complete opacification of the right hemithorax associated with mild compressive atelectasis.

**Fig. 2:**
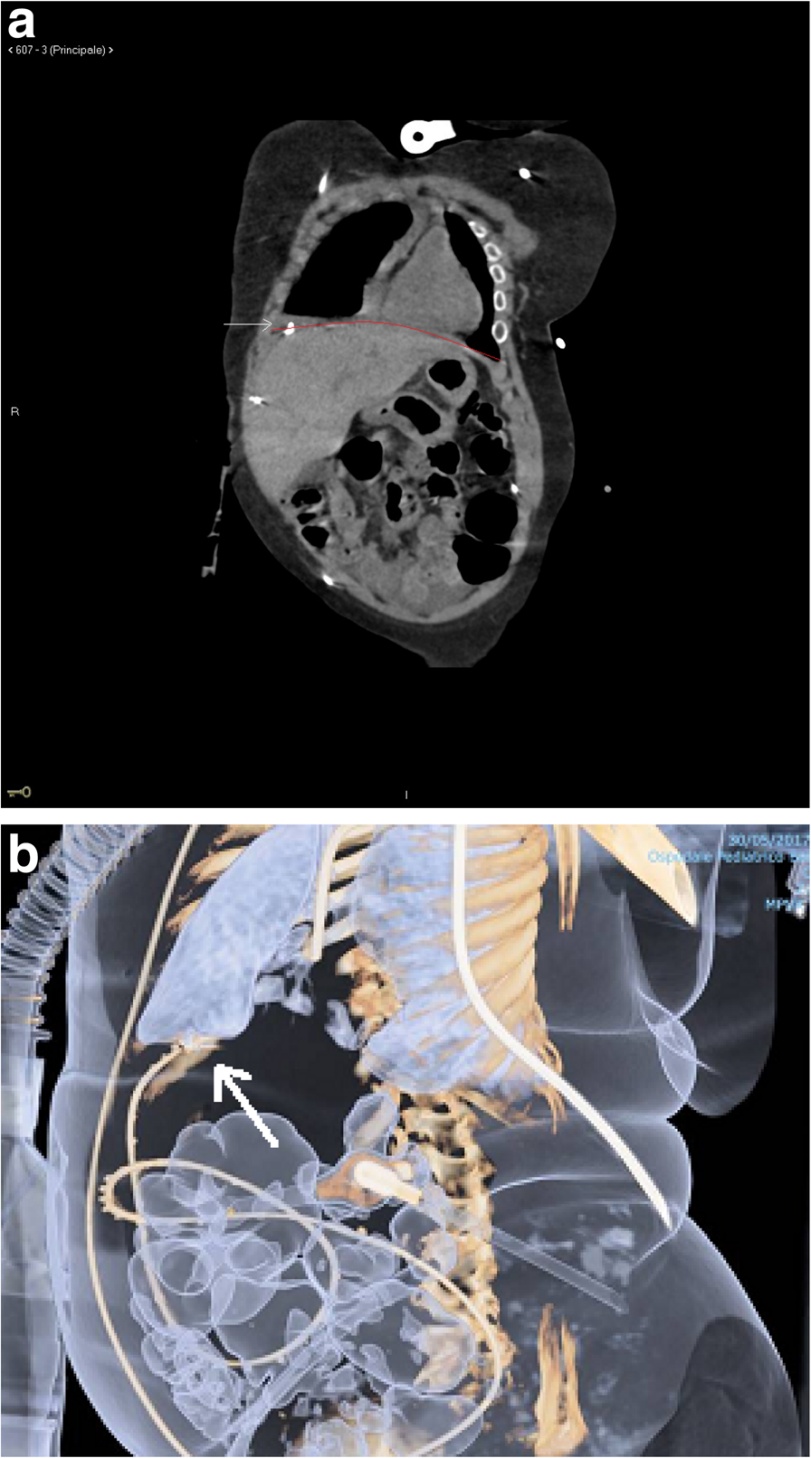
**a** Coronal plane of chest and abdomen CT scan demonstrating the dislocation of the distal end of VP shunt situated over the diaphragmatic cupola and within the pleural cavity. **b** Abdomen CT scan 3D reconstructions

A large right-sided pleural effusion was detected performing a chest CT scan 12 h after the admission. In addition, abdomen CT scan indicated the dislocation of the distal tip of the VP shunt located onto the diaphragmatic cupola within the pleural cavity (Fig. 2).

Because of the huge amount of the pleural effusion, diagnostic and therapeutic thoracentesis was immediately performed and a clear and colorless pleural fluid suspicious for CSF was aspirated and collected for analysis. Physical and chemical examination of the pleural fluid showed normal count of nucleated leukocytes (20/mm^3^) with content of glucose, protein and LDH respectively of 80 mg/dl, 0.40 g/dL and 79 IU/L; normal values of cholesterol (3 mg/dl) and triglycerides (6 mg/dl) were also detected. Beta-2 transferrin – a protein specific for CSF and perilymph – was found confirming a CSF leakage into the pleural cavity.

Since right pleural effusion recurred 7 days after and the hydrocephalus was not suitable for an endoscopic treatment, VP shunt was revised and shortened while a VA shunt was placed on the left side in order to serve as the main shunt and to prevent pleural effusions (Additional file 1). The decision of insertion of VA shunt on the left side was made because the right jugular vein was completely occluded. The valve system of the right VP shunt was also set to a very high opening pressure in order to start working only in case of VA shunt malfunction.

A new chest radiograph revealed the resolution of the pleural fluid collection and the expansion of the right lung. After thoracentesis, patient’s clinical condition immediately improved with normalization of respiratory rate and good oxygen saturation in room air.

Durable radiologic and clinical disease stability was obtained after neurosurgical procedures.

## Discussion

Complications of shunts are more frequently due to mechanical failures and infections [4]. Malfunction due to infection occurs approximately in 5 to 15% of treated patients [5]. Conversely, mechanical failure is the most frequent cause of shunt malfunction occurring during the first year after shunt placement [6]. Sometimes, anyway, shunts may be responsible for complications not due to their function but to the migration into undesired compartments.

**Fig. 3:**
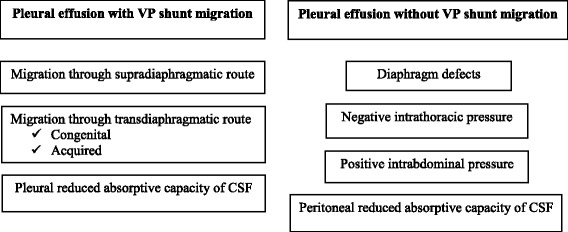
Factors contributing to CSF hydrothorax with or without intrathoracic VP migration

CSF pleural effusion in VP shunted patients may be associated or not to shunt displacement (Fig. 3). Over 60% of CSF hydrothorax cases are due to distal catheter tip migration into the thorax [7] and they are predominantly described in the paediatric population [8].

VP shunts may migrate in the thoracic cavity by either a supradiaphragmatic route (created at surgery during the passage of the shunt along the subcutaneous tissue) or transdiaphragmatic hiatuses (foramen of Bochdalek or Morgagni). Furthermore, authors hypothesize that local inflammatory reactions induced by the shunt tip may contribute to the diaphragm erosion facilitating shunt migration [3]. Once the shunt tip enters the pleural cavity, an effusion occurs when the CSF drained exceeds the absorptive capacity of the pleura. Because of children younger than 5 years have a small pleural surface area, pleural effusion is the most frequent presentation of VP intrathoracic displacement in the pediatric age [3].

Nevertheless, patients with VP catheter can present with pleural effusions even if the shunt tip remains in the peritoneal cavity [1, 9, 10]. In this case, diaphragm defects and/or decreased ability for peritoneal fluid absorption of CSF can facilitate the flow of fluids from the peritoneal cavity to the pleural one [1]. The negative intrathoracic pressure and the positive intrabdominal pressure contribute to the fluid shift.

Dyspnea is the common presenting symptom when pleural effusion is established, although tension hydrothorax with shock symptoms [11] and pleuritic chest pain have also been described [9, 12]. Nevertheless, respiratory complaints may develop gradually in adults because pleural absorption of CSF is not as limited as that in young children [10].

Novel diagnostic strategy has been proposed in order to define the source of the pleural effusion and the detection of beta-2 transferrin on pleural fluid is considered a specific marker for CSF leakage [13].

The injection of radioactive contrast dye at a point along the shunt course followed by imaging studies has also been used to study the course of VP shunt and investigate the source of pleural effusion [9]. Peritoneal scintigraphy, CT peritoneography and MR peritoneography using contrast/dye materials are useful when a peritoneal-thoracic fistula is suspected [14].

With this paper, we propose a pediatric case report of pleural effusion in VP shunted patient and a review of the literature pertaining to this rare complication.

We performed our search using the following MeSH terms: ventriculo-peritoneal catheter OR ventriculo-peritoneal shunt AND pleural effusion. The limit of pediatric age (0–18 years) has also been considered and all papers published on Pubmed have been included (Table 1).

**Table 1:** Summary of pediatric cases of pleural effusion secondary to VP shunt insertion published on PubMed

Authors	Age	Mechanism	Treatment	Interval From VP Shunt Insertion
Hadzikaric N [1]	16 mo	No intrathoracic migration	Thoracentesis, VA derivation positioning	2 mo
Akyuz M [7]	12 ys	Intrathoracic migration	Shunt review	5 mo
Faillace WJ [9]	4 mo	No intrathoracic migration	Thoracentesis, VA positioning	1 mo
Dickman CA [11]	1 mo	Intrathoracic migration	Thoracentesis	5 days
Gupta AK [15]	NA	NA	NA	NA
Martínez-Lage JF [16]	9 ys, 5 ys	NA	NA	NA
Ratliff M [17]	4 ys	No intrathoracic migration	Thoracentesis	1 day
Glatstein MM [18]	10 ys	Intrathoracic migration	Thoracentesis	10 ys
Samdani AF [19]	13 ys	Intrathoracic migration	Thoracentesis, VP shunt replacement	13 ys
Di Roio C [20]	20 mo	Intrathoracic migration	Thoracentesis, shunt review	20 mo
Cooper JR [21]	7 mo	Intrathoracic migration	Thoracentesis, shunt review	21 days
Kiran NA [22]	9 mo	Intrathoracic migration	Thoracentesis	3 mo
Ergun R [23]	5 mo	Intrathoracic migration	VP shunt replacement	3 mo
Karapolat S [24]	7 ys	Intrathoracic migration	Thoracothomy, VP shunt replacement	2 mo
Martin LM [25]	3 ys	Intrathoracic migration	Thoracentesis, shunt review	8 mo
Çakin H [26]	5 mo	Intrathoracic migration	Shunt review	5 mo
O’Halloran PJ [27]	5 ys	No intrathoracic migration	Thoracentesis, shunt review, VA derivation positioning	2 ys
Chuen-im P [28]	5 ys	No intrathoracic migration	Thoracentesis, pleurodesis, intracranial endoscopic choroid plexus coagulation	5 ys
Kocaogullar Y [29]	5 ys	No intrathoracic migration	Thoracentesis, VP removall,VA derivation positioning	4.7 ys
Born M [30]	2.5 ys	No intrathoracic migration	Thoracentesis, shunt review	1.5 ys
Adeolu AA [31]	8 ys	No intrathoracic migration	Shunt review	2.5 mo
Smith JC [32]	14 mo	No intrathoracic migration	Thoracentesis, shunt externalization	2.5 mo

Legend: NA, not available

Overall, 22 studies have been collected. Data about patient age is not available in one study [15]. Information about the interval between VP shunt insertion and the onset of pleural effusion, mechanism and treatment are not available for two papers [15, 16]. Patients’ ages range from 1 month to 13 years, and the interval between the original shunt insertion and the presentation with pleural effusion ranges from 5 days and 10 years. Only one case describes the occurrence of left pleural effusion 1 day after the insertion of left ventriculo-pleural shunt in a 4-year-old boy with complex history of post-hemorrhagic hydrocephalus and VP and VA shunt failure [17]. The intrathoracic migration of VP shunt is reported in 11/22 articles [7, 11, 18–26]; conversely, 9/21 papers describe the correct placement of VP shunt in the peritoneal cavity [1, 9, 17, 27–32]. Thoracentesis is the most frequent procedure used to diagnose and treat pleural effusions [1, 9–11, 17–30, 32]; thoracotomy has been performed in only one case [24]; bipleural drainage has been placed in one patient with pleural effusion due to ventriculo-pleural shunt positioning [17]. The review of distal tip of VP shunt and VP replacement have been described in 7 [7, 20, 21, 25–27, 30, 31] and 2 reports, [19, 23, 24] respectively. One patient underwent a VP shunt externalization [32]. VP shunt removal and VA shunt positioning have been reported for 4 patients [1, 9, 27, 29]. Intracranial endoscopic choroid plexus coagulation (CPC) has been required for 1 patient in which also pleurodesis failed to prevent recurrence of pleural effusion [28].

So, it is evident that thoracentesis is a useful tool to treat massive pleural effusion other than to define its source. Revision of the distal tip of the VP catheter may be enough when malfunction is suspected, especially when the effusion is not massive and clinical picture does not suggest catheter infection. Its removal from the pleural space and repositioning back into the peritoneal cavity or in the right atrium is considered when dislocation is showed or CSF hydrothorax is recurrent [33].

Positive pressure ventilation is also reported as a possible tool for the treatment of pleural effusion because of its effect on conversion of negative intrathoracic pressure to positive, preventing the fluid shifts [34].

## Conclusions

Our case has represented the opportunity to discuss a rare thoracic complication of VP catheter in patients with hydrocephalus. Even if CSF hydrothorax is rarely described, it should be suspected when respiratory failure, mechanical ventilation or oxygen requirement and persistent pleural effusion are detected in VP shunted patients in which no adequate neurological examination can be carried on. Beta-2 transferrin assay and radionuclide shuntography are useful techniques to diagnose CSF pleural effusion and verify the shunt patency and course. The diagnostic work up should include investigations excluding peritoneal-thoracic fistula. Thoracentesis and shunt revisions are common; different types of CSF shunting (VA shunt) or endoscopic treatment (third ventriculostomy, ETV, associated to choroid plexus coagulation, CPC) may be considered as alternative therapeutic approaches.

## Additional file

Additional file 1: Timeline. (DOCX 28 kb)

## Abbreviations

CPC: Choroid plexus coagulation
CSF: Cerebrospinal fluid
CT: Computed thomography
ETV: Endoscopic third ventriculostomy
LPM: Liters per minute
VA: Ventriculo-atrial shunt
VP shunt: Ventriculo-peritoneal shunt
