# Supplementary figures and images for: Pleural effusion from intrathoracic migration of a ventriculo-peritoneal shunt catheter: pediatric case report and review of the literature
Source: Ital J Pediatr. 2018 Mar 27;44:42. doi: 10.1186/s13052-018-0480-2 (PMC5870185; doi:10.1186/s13052-018-0480-2)

**
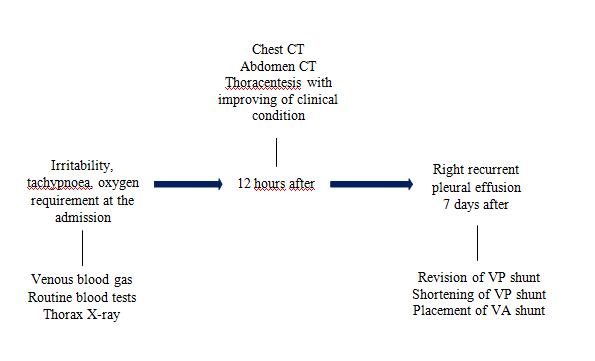
**

Supplement: Supplementary file 1 — Timeline. (DOCX 28 kb) [file 13052_2018_480_MOESM1_ESM.docx]
